# Poor Accrual in Palliative Research Studies: An Update From the Rapid Response Radiotherapy Program

**DOI:** 10.4021/wjon357w

**Published:** 2011-10-28

**Authors:** Karen Lien, Liang Zeng, Nicole Bradley, Shaelyn Culleton, Marko Popovic, Julia Di Giovanni, Rehana Jamani, Gemma Cramarossa, Janet Nguyen, Kaitlin Koo, Florencia Jon, Edward Chow

**Affiliations:** a Rapid Response Radiotherapy Program, Department of Radiation Oncology, Odette Cancer Centre, Sunnybrook Health Sciences Centre, University of Toronto, Canada

**Keywords:** Poor accrual, Palliative care, Research studies, Attrition

## Abstract

**Background:**

In June 2003, the Rapid Response Radiotherapy Program (RRRP) implemented changes to recruitment strategies in attempts to increase patient accrual to research studies. Such modifications included the use of a dedicated research assistant to screen for and identify eligible study patients, the introduction of more appropriate inclusion criteria, and the switch towards telephone interviews to minimize patient burden. The purpose of this study is to provide an update on patient accrual in the RRRP.

**Methods:**

All patients seen in the RRRP from January 2002 to December 2009 were recorded in a prospective database. Reasons for referral, eligibility for clinical trials, reasons for non-accrual, and various demographics information were recorded. Descriptive statistics summarized findings.

**Results:**

A total of 4726 patient visits were recorded from January 1st, 2002 to December 31st, 2009. Prior to changes, the overall rate of accrual into research studies was 14.9% versus 48.1% after changes were implemented. Patients were not accrued onto studies mainly to due ineligibility according to study protocol. Other reasons such as language barrier (12.1%), physician objection (3.5%), patient declining participation (11.3%) and lack of a research assistant (9.3%) were cited.

**Conclusions:**

Changes in clinical structure and study design can significantly impact accrual patterns in palliative radiotherapy studies. Despite these changes however, the majority of patients are still not enrolled in studies. Therefore additional efforts need to be made to maximize patient accrual and minimize attrition.

## Introduction

In 2011, it is projected that approximately 177 800 new cases of cancer and 75 000 cancer related deaths will occur in Canada [1]. As cancer progresses to a point where cure is no longer possible, palliative or end-of-life care are initiated with the goal of improving quality of life. As part of the Canadian Strategy for Cancer Control, palliative care has been deemed one of the five priorities of health care; but until recently, literature has been relatively sparse for end-of-life care [2, 3]. Studies have cited difficulties in defining fundamental concepts, such as quality of life (QOL) and death, as significant challenges faced while conducting this research [2]. For palliative research that has been published, authors often described the immense difficulty in patient accrual and high rates of attrition for this select population [2, 4-8].

Significant barriers to conducting palliative care studies exist as a result of the unique challenges that arise when working with advanced cancer patients. Patient participation has been known to be extremely low due to family concerns and limited life expectancy, and the implications of such significantly affect the availability and quality of research in the palliative patient population [4, 6-12]. Trials are often closed prematurely due to the difficulty in reaching appropriate sample sizes. Even if patients are accrued to studies, the rates of attrition are significant, with some studies reporting up to a 60% patient dropout rate [9]. In addition, many have even questioned the ethics of conducting research in a potentially vulnerable group of patients, where cognitive impairment or deteriorating cognitive function may impede the informed consent process [10].

Clinical trials allow health care professionals to provide patients with quality, evidence-based, timely care that reflects the most recent data. These trials often exhibit additional methodological concerns when involving an advanced cancer population but are needed to establish best practice guidelines and to make advancements and improvements in the palliative care setting [9]. Evidence-based data result in the production of new, more efficacious methods for treating patients and can help to best optimize resources. For example, palliative radiotherapy randomized controlled trials in patients with bone metastases have shown that radiation dosages of 20 Gy in 5 fractions when compared to 8 Gy in 1 fraction are equally efficacious with respect to pain relief [13]. This information encourages radiation oncologists to prescribe fewer fractions in already fragile patients seeking pain relief, thereby reducing patient burden and optimizing resource utilization.

Although research is becoming more prevalent in palliative care, studies are still affected by poor accrual, and high attrition rates continue to be a concern for research quality. The Rapid Response Radiotherapy Program (RRRP) at Sunnybrook Odette Cancer Centre provides timely access to palliative radiotherapy for advanced cancer patients with the goal of symptom management. A previous report by our group analyzed the patient accrual patterns in the RRRP between January 2002 and December 2004 [4]. In May 2003, changes were made which included the addition of a research assistant, along with augmented study inclusion criteria and the addition of patient telephone follow-ups. This initial study analyzed pre- and post-May 2003 accrual patterns and demonstrated a marked improvement in patient accrual once changes were made [4].The purpose of this subsequent study was to update and incorporate additional accrual data from a significantly larger time period (January 2005 to December 2009) to better evaluate and discuss the factors associated with poor accrual in this patient population.

## Methods

The RRRP at the Sunnybrook Odette Cancer Centre was established in 1996 as a pilot project to provide quick access palliative radiotherapy for advanced cancer patients. Since its inception, patients referred to the RRRP have often been seen within a week of referral, which is a significant improvement over the acceptable Canadian wait time of 17 days for similar treatment [14]. The RRRP clinic runs daily each morning, and is staffed by a radiation oncologist, nurse, radiation therapist and research student. Palliative research studies in the RRRP are ongoing specific to radiotherapy intervention, treatment outcomes, or other quality of life (QOL) issues in the palliative cancer patient population. As of writing, there were eight ongoing studies each with varying eligibility criteria and follow-up periods.

Prior to June 2003, study accrual in the RRRP was conducted by research radiation therapists or nurses who also handled varying amounts of clinical responsibilities. With an increase in patients referred, clinicians were becoming overwhelmed with clinic responsibilities, resulting in a decrease in study recruitment. A full-time research assistant was hired in June 2003 to take over the responsibility of research coordination, resulting in increased accrual from approximately 14% from January 2002 to May 2003 to 60% after hiring a dedicated research assistant [4]. This study, however, included a limited amount of patients, with 483 patients prior to June 2003 and 712 patients post June 2003 [4].

A clinical database has been maintained since 2002 to document patients who have visited the RRRP and has collected their basic referral and patient demographic information including: age, gender, primary cancer, and reason for referral. Information has also been collected regarding patient study participation and reasons for non-accrual. A research assistant is given permission by the radiation oncologist or therapist in clinic to introduce the study to the patient based on a variety of factors including: study inclusion criteria; performance status; likelihood of completing follow up; emotional and psychosocial stability; and any other factors which may impact study participation. If a patient then declines participation or is not a suitable candidate for a research study, a reason, if applicable, is recorded. Not all patients seen in the RRRP were eligible for study participation, but their data was collected and entered into a prospective clinical database. Patients participating in any studies provided informed consent and were subsequently recorded in the clinical database as an accrual.

### Palliative study design

Study design and eligibility criteria are known barriers in patient accrual for clinical research studies, in particular, for research being conducted in a palliative care setting. As a result, research studies implemented in the RRRP were created to involve mainly concise, simple QOL questionnaires that are relevant to the majority of patients referred for our services. For example, two major studies at present involve validated QOL questionnaires that examine outcomes after radiotherapy in patients with bone and brain metastases. These require patients to complete a concise, simple 10-minute questionnaire at baseline and repeat that same questionnaire during follow-up over the telephone. Since the majority of the cases seen at the RRRP are for symptomatic bone or brain metastases treatment, a large percentage of referrals are eligible for the clinic’s research studies. In addition to using simple questionnaires that are appropriate for the palliative cancer population, studies that require collection of biological samples are structured to minimize patient burden. Urine and saliva samples are used in place of blood samples to make baseline and follow-up collection easier and less invasive for patients. Protocols are often created with brevity and minimal patient impact in mind in order to increase accrual and successful study completion.

### Statistical analyses

Descriptive statistics were used to summarize data collected from all patients attending the RRRP from January 2002 to December 2009. The time periods were split into ‘prior to research changes’ and ‘after research changes’; descriptive statistics detailed changes between these two time periods.

## Results

A total of 4796 patient visits were recorded for the period from January 1st, 2002 to December 31st, 2009. Before research changes were made, a total of 533 patients were seen in the RRRP spanning January 1st, 2002 through to May 31st, 2003. After changes were implemented, a total of 4243 patients were seen from June 1st, 2003 to December 31st, 2009. Table 1 lists patient demographics from both these groups, including reasons for referral and primary cancer sites. The average age of patients attending the RRRP was pre and post research changes was 67 and 68 years respectively with the majority coming from home (pre: 70.8%; post: 70.4%). Primary cancers of the lung, breast, and prostate were most common. Patients were referred mostly for bone pain (pre: 54.6%; post: 53.5%) followed by brain metastases (pre: 24.1%; post: 22.3%).

**Table 1:** Patient Demographics

	Before Research Changes	After Research Changes
**n (Number of patients)**	483	4243
**Sex**		
Male	254 (52.6%)	2240 (52.8%)
Female	229 (47.4%)	2003 (47.2%)
**Age at initial consultation (years)**		
Mean ± standard deviation	66.8 ± 12.1	67.9 ± 12.7
Median (Range)	68 (23-95)	69 (21-101)
**Patient came from**		
Home	369 (76.4%)	2986 (70.4%)
Hospital or hospice	109 (22.6%)	1084 (25.5%)
Unknown	5 (1.0%)	173 (4.1%)
**Ambulance**^a^		
Yes	108 (23.4%)	991 (23.4%)
No	375 (77.6%)	3173 (74.7%)
Unknown	-	79 (1.9%)
**Primary cancer site**		
Lung	171 (35.4%)	1493 (37.3%)
Breast	95 (19.7%)	894 (22.3%)
Prostate	80 (16.6%)	701 (17.5%)
Gastrointestinal	50 (10.4%)	333 (8.3%)
Unknown primary	30 (6.2%)	265 (6.6%)
Renal cell	23 (4.8%)	217 (5.4%)
Others	34 (7.0%)	105 (2.6%)
**Reason(s) for referral**^b^		
Bone pain	302 (54.6%)	2355 (53.5%)
Brain metastases	133 (24.1%)	980 (22.3%)
Mass	34 (6.2%)	262 (6.0%)
Shortness of breath	23 (4.2%)	117 (2.7%)
Other Pain	34 (6.1%)	55 (1.3%)
Assess previous palliative Radiotherapy response	16 (2.9%)	133 (3.0%)
Other reasons	36 (6.5%)	141 (3.2%)
Need for more radiation treatment	27 (4.9%)	55 (1.3%)
Spinal cord compression or cauda equina compression	19 (3.4%)	76 (1.7%)
Bleeding	13 (2.4%)	92 (2.1%)
Pathological rracture	16 (2.9%)	55 (1.3%)
Impending spinal cord compression	12 (2.2%)	37 (0.8%)
SVCO Symptoms	2 (0.4%)	42 (1.0%)

a: Some patients were not seen by research students due to various reasons; b: Some patients may have been referred for >1 reason; SVCO: Superior Vena Cava Obstruction.

**Figure 1:**
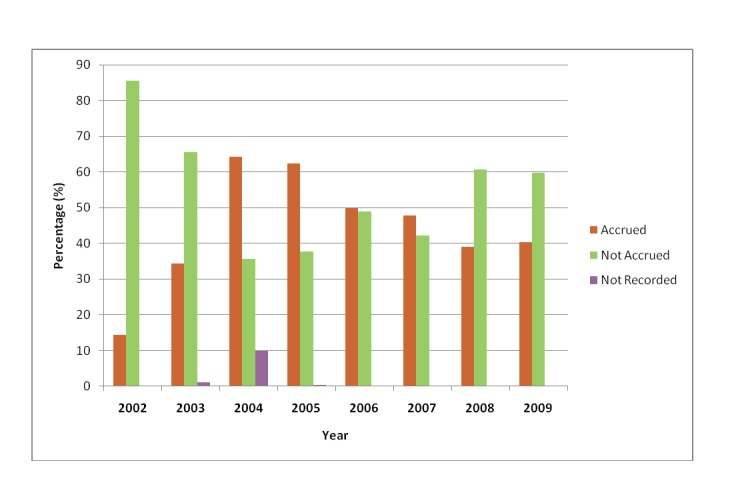
Annual accrual rates from 2005-2009.

Prior to changes in research practices, patient accrual was approximately 14%. After research changes, just under half of patients (48%) were being accrued into studies which represents a 34% improvement. Results pertaining to accrual can be found in Table 2; Figure 1 details annual accrual rates from 2002-2009. In both research time periods, the primary reason for non-accrual was patient ineligibility (pre: 35.6%; post: 42.1%). Other reasons such as language barrier (pre: 6.8%; post: 14.1%), physician objection (pre: 3.0%; post: 4.9%), patient declining participation (per: 3.0%; post: 10.9%) and lack of a research assistant (pre: 1.5%; post: 9.1%) were cited. It should be noted that with data collected prior to research changes, a total of 45.3% of patients had no reason recorded for non-accrual when compared with just 3.3% after research changes. This makes comparisons of non-accrual reasons between the two time periods difficult to assess.

**Table 2:** Reasons for Non-Participation

	Before Research Changes	After Research Changes
**Number of patient encounters in RRRP**	553	4243
**Number of non-accrued patient encounters in RRRP clinic**	474 (85.7%)	2202 (51.9%)
**Reasons for Non-accrual^A^**		
Patient ineligible according to study protocol	171 (35.6%)	918 (42.1%)
Language barrier	32 (6.8%)	307 (14.1%)
Physician objection	14 (3.0%)	107 (4.9%)
Patient mentally incapable of participating due to cognitive deterioration and inability to provide informed consent or complete assessments	14 (3.0%)	110 (5.0%)
Patient declined participation	14 (3.0%)	237 (10.9%)
Patient too ill or unwell	7 (2.0%)	169 (7.7%)
No research assistant available at time of consultation or too busy to recruit patients	7 (1.5%)	199 (9.1%)
Patient too emotional or anxious about illness or treatment	5 (1.1%)	56 (2.6%)
Patient drowsy	1 (0.2%)	7 (0.3%)
Unknown reason (not recorded)	215 (45.3%)	72 (3.3%)

A: Patients may have more than one reason for non-participation.

## Discussion

Research studies are essential for improving treatment options and advancing patient care. However, various factors such as rapid disease progression and cognitive deterioration contribute to unique challenges of patient recruitment to research studies and high attrition rates in advanced cancer patients. In June 2003, the RRRP implemented changes to study design and recruitment processes, resulting in significant improvements in accrual for subsequent studies. In providing an update on participation in RRRP research studies, additional strategies have been identified to further improve recruiting strategies, increase accrual and limit the effects of high attrition.

Difficulty enrolling advanced cancer patients onto clinical trials may be due to reluctance by health care practitioners themselves. Gate keeping is described as healthcare professional (HCP) originated reluctance to accrue patients onto studies [15] and may occur for a number of reasons. Henderson et al. felt that HCP themselves may influence accrual rates for palliative care studies through the thought of protecting very vulnerable patients from demanding research projects [7]. White et al. [15] ADDIN RW.CITE{{12 White,C. 2008}} distributed a questionnaire to palliative HCPs regarding willingness to participate in randomized controlled trials (RCTs). This study reported that a majority of HCPs would refer their patients to non-pharmacological studies but were much more reluctant to do so for pharmacological studies where patients might have adverse side effects [15, 16]. Typically, studies that are brief and concise or involve symptom control are favored [15]. As very much a reflection of White et al.’s [15] findings, studies conducted in the RRRP are designed to be of minimal burden on patients and involve questionnaires regarding QOL that take approximately 10-15 minutes at baseline and follow-up complete.

White et al. also identified previous research exposure and experience as a significant predictor for willingness to refer patients for participation in RCTs [15]. This could help explain the low rate of physician objection to accrual at our center due to experience in conducting palliative care research. However with additional experience, investigators may adjust their attitudes towards accrual of specific subsets of patients that are very difficult to consent and follow. For example, one major problem affecting the participation rates for the ‘post research changes’ study period was the fact that primary investigators no longer wished to accrue inpatients onto studies. From previous experience, it was found that inpatients, especially those from other hospitals, were too difficult to contact for follow-up. As a result, attrition was extremely high for that group. This may explain the increase in non-accrual due to ineligibility and also possibly the reason for decrease in accrual rate when compared with our group’s initial report (59.4% from June 2003-December 2004 versus 49.0% from January 2005-December 2009).

During May 2002 to August 2004, an in-house trial at the RRRP was held to examine the effectiveness of whole brain radiotherapy using QOL assessments. As part of this study, patients were required to physically attend the RRRP to complete the follow up study assessments. Patient accrual was limited as the majority of patients refused to participate due to the inconvenience and potential challenges of travelling to the clinic to fill out a follow-up questionnaire for patients with limited life expectancy. After this experience, more patient-friendly follow up methods and the options of telephone, fax, email or mail-in questionnaires were adopted to reduce patient burden. This modification in study design was significant in reducing patient burden and allowed for increased accrual in subsequent studies. A recurrent theme of minimizing patient burden is crucial to improve accrual rates for patients who are deemed eligible for study inclusion. Our use of telephone follow-up interviews has been supported as a means to collect longitudinal data for patients, as well as to maintain a high level of researcher-patient rapport [17]. Another alternative may be to schedule follow-up interviews and assessments into upcoming clinical visits if possible [18]. As the availability of technology grows, other options such as interactive voice response systems (IVRS) may be used to collect follow-up data. Such an approach would involve automated telephone reminders to complete assessments and would allow patients to complete assessments when is best convenient for them, rather than waiting until both the research assistant and patient’s schedules are agreeable [19].

Study coordinators have realized the difficulty in collecting data in palliative patients and therefore studies are designed and coordinated to be as patient friendly as possible. When examining various aspects of QOL, questionnaires are designed to be as short as possible. For example, condensed QOL assessments such as the EORTC-QLQ-C15-PAL can be used as an alternative to the standard EORTC-QLQ-C30 assessment ADDIN RW.CITE{{45 Groenvold,M. 2006}}[20]. Steinmann et al. compared the practicality of both EORTC assessments in those with brain metastases in a pilot study and found that centers preferred the shorter version and that patient compliance using the shorter assessment was better [21]. Now in the main phase of their study, all centers are using the condensed version of the QOL assessment [21]. Not only should assessments be short in length, but care should be taken to ensure that patients understand the questions asked of them, as poorly understood items commonly result in unanswered questions and missing data [22]. Occasionally, assessments utilize words and concepts that may be synonymous with others or are poorly defined. To ensure some degree of standardized answers, research personnel should be consistent in explaining study procedures to patients.

Involvement of the family or caregiver represents an important aspect in both accrual and subsequent data collection for study patients. Caregivers may also act as gatekeepers, objecting to study involvement for their family members [11]. For this reason, the informed consent process should be geared towards establishing a good relationship between patients, their family and caregivers. Also, as patients deteriorate, the family or caregiver will play a prominent role in proxy data collection [6]. The collection of proxy data represents a promising means to overcome significant drop-offs of data points for patients too ill to complete assessments, but patient-proxy agreement seems to vary by population and assessment tools [23-25]. Nevertheless, caregiver and family involvement is an important aspect of patient accrual. Steinhauser et al. sought to enroll a caregiver for each patient in study and suggested that through engaging the caregiver as a participant, patient recruitment improved and the perception of patient burden actually decreased [17]. Additionally, a number of studies recommend small gestures, such as personalized thank you cards or certificates of appreciation, to establish researcher-patient rapport, as these efforts may reduce attrition by encouraging patient participation in studies [6, 17].

It is important for investigators to consider expected attrition, as previous studies recommend performing sample size calculations that incorporate the effects of high attrition rates [6, 18]. Mitchell et al. performed a comparison of methodologies used by two different studies involving similar palliative care interventions and found that it was advantageous to inflate the sample size to account for attrition [18]. The study that did not adjust their sample size to account for attrition employed a strategy of vigorous screening and gatekeeping in order to recruit ‘ideal’ patients. However, despite such vigorous screening methods and an inclusion criteria that included a minimum life expectancy greater than one month, over 50% of recruited patients died or withdrew before the one month follow-up and the study was not able to reach target sample size. A number of factors were identified that helped the first study reach target accrual such as: increased funding; eligibility that included maximal inclusion and minimal exclusion criteria; and specific recruitment strategies gleaned from the literature which were subsequently adapted for use in the palliative setting. Such recruitment strategies included the use of a dedicated research nurse, significant marketing to raise awareness of the study, as well as the minimization of gate keeping by assigning tasks such as 48-hour survival assessment to an independent nurse. The results of this study indicate that good accrual is possible but is largely dependent on the funding available to undertake various recruitment strategies.

Over the updated period of time, we saw a significant increase in the number of patients who were ineligible and did not meet study inclusion criteria for various reasons. Depending on the studies available at the time as well as the specific patient group they targeted, it is expected that the eligible number of patients would fluctuate over this period. Furthermore, during this period of time, we saw an increased rate of inappropriate referrals. As more of these patients were directed to the RRRP, accrual rates would be negatively impacted as these patients would not be treated or accrued onto a research study, but would instead be referred to the other clinics or for further investigations. As poor performance status and cognitive function are commonly used as exclusion criteria for studies in the palliative setting, this also may have artificially inflated the number of patients not accrued due to ineligibility [26]. Despite exclusion of patients with poor cognitive or performance status, a study by Petersen et al. found only modest differences between participants and non-participants on QOL assessment items [27]. While status or cognition of patients in clinic cannot be predicted or controlled, a concerted effort should be made to accrue patients in studies with a more inclusive study criterion to reduce ineligibility.

There are several limitations to the ‘reason for non-accrual’ data collected for this study. Since June 2003, the RRRP has several research assistants and thus varying interpretations of ‘reason for non-accrual’ could occur. In addition, ‘reason for non-accrual’ data was rarely recorded prior to June 2003, making comparisons between pre and post research changes more difficult to interpret. Patients could also have multiple reasons for non-accrual, and only one or a few of them were recorded in the database.

Overall, clinical trials are essential for advancing the field of palliative care and to improve treatment options and delivery for patients. Poor accrual and attrition significantly impact many studies by weakening the quality of results or by leading to study termination. Efforts should be taken to maximize accrual, as this represents a way to reduce the effects of attrition on data quality. Additionally, high rates of attrition should be addressed by incorporating study design that decrease patient burden. Although the literature contains many studies in which poor accrual and attrition were limiting factors, it is also rife with strategies on how to approach such problems. In 2003, the RRRP made significant changes to recruitment strategy and saw positive results with respect to the percentage of patients accrued to study. These changes included the addition of a dedicated research assistant to the clinical team, as well as the modification of to study design to be minimally burdensome for patients. At present, we report that the changes undertaken previously are sustainable and still in place, while new recruitment attitudes and strategies have been shaped by prior experience. Future studies should continue to maximize accrual through various strategies including the following: involvement of family and caregivers throughout the informed consent and data collection processes, use of concise assessments, accounting for attrition in sample sizes, and employment of study marketing to increase awareness of available studies.
